# Associations of metabolic syndrome and diabetes mellitus with 16-year survival after CABG

**DOI:** 10.1186/1475-2840-13-25

**Published:** 2014-01-22

**Authors:** Ville Hällberg, Ari Palomäki, Jorma Lahtela, Seppo Voutilainen, Matti Tarkka, Matti Kataja

**Affiliations:** 1 Kanta-Häme Central Hospital, Hämeenlinna, Finland; 2 University of Tampere, Medical School, Tampere, Finland; 3 Tampere University Hospital, Tampere, Finland; 4 Päijät-Häme Central Hospital, Lahti, Finland; 5 National Institute for Health and Welfare, Helsinki, Finland

**Keywords:** Coronary artery bypass grafting, Diabetes mellitus, Metabolic syndrome, Follow-up, Mortality

## Abstract

**Background:**

The associations of metabolic syndrome (MetS) or diabetes mellitus (DM) on long-term survival after coronary artery bypass grafting (CABG) have not been extensively evaluated. The aim of the present study was to assess the impact of MetS and DM on the 16-year survival after CABG.

**Methods:**

Diabetic and metabolic status together with relevant cardiovascular data was established in 910 CABG patients operated in 1993-94. They were divided in three groups as follows: neither DM nor MetS (375 patients), MetS alone (279 patients) and DM with or without MetS (256 patients). The 16-year follow-up of patient survival was carried out using national health databases. The relative survival rates were analyzed using the Life Table method comparing the observed survival rates of three patient groups to the rates based on age-, sex- and time-specific life tables for the whole population in Finland. To study the independent significance of MetS and DM for clinical outcome, multivariate analysis was made using an optimizing stepwise procedure based on the Bayesian approach.

**Results:**

Bayesian multivariate analysis revealed together six variables to predict clinical outcome (2 months to 16 years) in relation to the national background population, i.e. age, diabetes, left ventricular ejection fraction, BMI, perfusion time during the CABG and peripheral arterial disease. Our principal finding was that after postoperative period the 16-year prognosis of patients with neither DM nor MetS was better than that of the age-, sex-and time-matched background population (relative survival against background population 1.037, p < 0.0001). The overall survival of MetS patients resembled that of the matched background population (relative survival 0.998, NS). DM was associated with significantly increased mortality (relative survival 0.86, p < 0.0001). Additionally, mortality was even higher in patients receiving insulin treatment than in those without. Excess death rate of DM patients was predominantly caused by cardiovascular causes.

**Conclusion:**

In this long-term follow-up study patient groups without diabetes had at least equal 16 years’ survival after CABG than their matched background populations. Survival of DM patients started to deteriorate already few years after the operation.

## Introduction

Metabolic syndrome (MetS), was first officially defined by the World Health Organization (WHO) and the National Cholesterol Education Program (NCEP) more than ten years ago [1,2]. Since that time its epidemiology has been widely evaluated [3,4]. MetS can be used as a tool to characterize patients at added risk [5-7]. Several studies have suggested that the risks of premature death and cardiovascular disease or diabetes are higher among subjects with MetS compared to those without. However, follow-up studies on long-term survival after coronary artery bypass grafting (CABG) have yielded controversial results with regard to the impact of MetS [8,9].

Diabetes (DM) is a prominent cardiovascular risk factor [10,11]. There is a considerable body of evidence on poor early outcome and higher in-hospital morbidity in diabetics compared with non-diabetic patients after CABG [12,13]. The association of diabetes on long-term survival after CABG has not been extensively evaluated. The few reports published comparing the type of treatment of diabetes to the long-term prognosis after CABG have come to conflicting conclusions [14,15].

Our aim was to evaluate the assumed detrimental impact of metabolic syndrome and diabetes mellitus on long-term prognosis after CABG, focusing on survival after the first two postoperative months.

## Methods

### Study population

**Figure 1:**
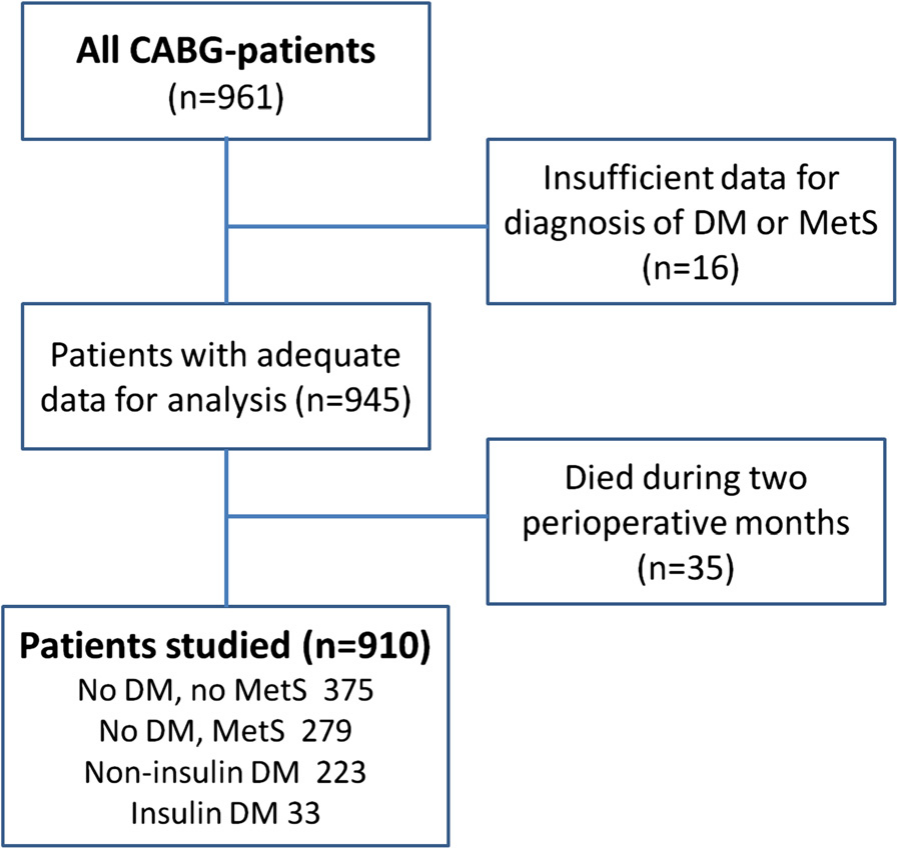
**Schematic description of study patients.** CABG = coronary artery bypass grafting, DM = diabetes mellitus, MetS = metabolic syndrome, Non-insulin DM = diabetic patients without insulin treatment. Insulin DM = diabetic patients treated with insulin.

The population of the Working after CABG (W-CABG) study has been described elsewhere [16]. Briefly, CABG was done on 961 patients in Tampere University Hospital during a period of 18 months in 1993-94. Data on MetS and DM were available on 945 operated patients and 910 patients survived two months after CABG (Figure 1). These patients formed the study cohort and were followed for 16 years.

We analyzed the survival of CABG patients in three groups as follows: neither DM nor MetS (DM-/MetS-), MetS alone (DM-/MetS+) and DM with or without MetS (DM+). We combined all DM patients in the same group regardless of their status concerning MetS, in primary analyses of diabetic patients the presence of MetS did not affect survival.

### Data collection and clinical definitions

The background of the patients and perioperative data were collected from the medical records in Tampere University Hospital and the secondary hospitals involved in the postoperative care. A survival analysis was made after the closing day, June 30, 2010, based on data obtained from the Statistical Office of Finland. The results of the full completed 16-year follow-up are given.

Diagnoses of hypertension, peripheral vascular disease, previous myocardial infarction, and previous transient ischemic attack (TIA) or stroke were established from patient records. Renal dysfunction was defined as a glomerular filtration rate less than 60 ml/min/1,73 m^2^ calculated with the CKD-EPI equation [17]. Patients were defined as hypercholesterolemic if they had total cholesterol ≥5 mmol/l (200 mg/dl) (NCEP ATP III) or were on lipid-lowering drug therapy. Smoking habits were based on a questionnaire completed 21 months postoperatively [16].

Classification of MetS was made according to the NCEP/ATP III definition with the exception that body mass index (BMI) (kg/m^2^) was used as a measure of obesity. During the 1990s waist circumference was not commonly measured [9,18]. Modified MetS was defined as the presence of three or more of the following risk factors: BMI > 30 kg/m^2^ for men and > 25 kg/m^2^ for women, TGL concentration ≥ 150 mg/dL (1.7 mmol/l), HDL-C < 40 mg/dL (1.03 mmol/l) for men and < 50 mg/dL (1.3 mmol/l) for women and systolic blood pressure ≥ 130 mmHg or diastolic blood pressure ≥ 85 mmHg or on antihypertensive medication. Elevated blood glucose was defined as fasting blood glucose ≥ 100 mg/dl (5.6 mmol/l) [1,2]. For the diagnosis of diabetes the 1998 WHO criteria were used, defining fasting blood glucose level (fB-gluk) ≥ 110 mg/dL (6.1 mmol/l) or random blood glucose ≥ 180 mg/dL (10.0 mmol/l) [1]. A patient was also defined as diabetic if on antidiabetic medication according either to hospital records or the reimbursement database maintained by the Social Insurance Institution of Finland. Further they were divided in insulin-treated diabetics regardless of oral antidiabetic medication and in non-insulin-treated diabetics based on hospital records at the time of admission for CABG. BMI was calculated as mass/height^2^ (kg/m^2^).

Cardiovascular death was defined as any death caused by coronary heart disease or sudden death during 2 months to 16 years postoperatively. Stroke was considered as cardiovascular death.

### Survival analysis

Overall survival (0-16 years postoperatively) was analyzed, including hospital and immediate postoperative survival for 945 patients. Separate survival analyses (2 months to 10 and 16 years) were then carried out for 910 patients, focusing on survival after the immediate postoperative phase. Survival analyses were made according to the metabolic status of the patients (DM-/MetS-, DM-/MetS+ and DM+). The cumulative relative mortality for each group was calculated in 2-month steps against age-, sex- and time-specific national background populations as described in Statistical methods. The first subanalysis was then made in similar manner to reveal survival of DM patients with and without insulin therapy. In the second subanalysis the association of MetS (yes or no) with survival was identically studied in DM patients. The study protocol was approved by the Ethics Committee of Tampere University Hospital.

### Statistical methods

In the comparison of patient characteristics, categorical data are tabulated as frequencies and percentages, and continuous variables are expressed as mean and SD. Differences between two groups were tested by Chi squared test and the Wilcoxon rank test, and in cases of more than two groups by Kruskal-Wallis test. Fisher’s exact test was used when appropriate in two-by-two tables with directed hypothesis. Differences in mean values between two groups were tested by Student’s t-test and in the case of more than two groups by analysis of variance.

The relative survival rate was analyzed by sex, age, DM and MetS using the Life Table method [19]. In this approach, the observed survival rates of the groups are compared to the rates based on age-, sex- and time-specific life tables for the whole population in Finland. Calculation of survival rates was based on the individual life expectancies of the target population for the target years (reference population). The survival of the reference population is effectively 1.00. If the survival curve of the group remains below that of the reference population there is excess mortality in the group.

To study the independent significance of MetS and DM for clinical outcome (2 months to 16 years), multivariate analysis was made using an optimizing stepwise procedure based on the Bayesian approach [20]. This procedure was developed for nominal variables, and does not require a perfect variable matrix. It selects, by the heuristic approach, the combination of variables which best explains the selected outcome factor. The Bayesian approach is applied by counting posterior likelihood ratios or odds ratios for each combination. The aim was to find an optimal subset of pre-and intraoperative variables to provide the best explanation. The parameters included in the multivariate analysis were age, gender, BMI, all significant cardiometabolic diseases and related operations as well as cardiac, lipid lowering and psychiatric medications. Also essential intraoperative characteristics of CABG were included in the analysis. Altogether 31 parameters were included in the Bayesian approach (Additional file 1: Table S1).

## Results

### Study population

The essential clinical characteristics of the 910 patients are shown in Table 1. Their mean age was 61.6 (SD 8.4) years; 191 of them were women (mean age 64.8 [SD 7.5] years) and 719 men (mean age 60.8 [SD 8.4] years). Of the 910 subjects, 41% were free of MetS or DM and 31% had MetS without DM (Figure 1).

**Table 1:** Preoperative demographic data, clinical characteristics and severity of coronary heart disease in 910 patients surviving two months after CABG

	**DM–/MetS–**	**DM–/MetS+**	**DM+**	**Overall**
	**n = 375**	**n = 279**	**n = 256**	**p-value**
**Female (%)**	15.2	26.2	23.8	<0.01^1,2^
**Mean Age (years) (SD)**	61.3 (8.2)	60.7 (8.6)	63.3 (8.4)	<0.001^2,3^
**Variables of Modified MetS**				
**BMI (kg/ m**^**2**^**)**	25.8	28.7	28.1	NA
**Elevated Blood Pressure (%)**	40.9	83.1	67.5	
**HDL Cholesterol (low, %)**	44.9	90.2	65.3	
**Triglyceride (high, %)**	27.4	83.7	67.7	
**Elevated Glucose or DM (%)**	4.5	25.8	100.0	
**Concomitant Diseases**				
**Hypercholesterolemia (%)**	69.5	80.3	77.7	NS
**Previous MI (%)**	70.5	67.8	67.7	NS
**Previous TIA or stroke (%)**	8.9	8.6	13.9	NS
**Intermittent Claudication (%)**	6.2	4.7	18.6	<0.001^3,4^
**Renal Function**				
**CreaCl (ml/min) (SD)**	76.4 (13.8)	74.2 (18.2)	73.7 (16.9)	NS
**CreaCl < 60 ml/min/1,73 m**^**2**^**(%)**	11.0	19.0	19.7	<0.01^1,2^
**Preoperative Smoking (%)**	66.6	60.2	66.4	NS
**Severity of Heart Disease**				
**EF (%) (SD)**	60 (14)	61 (13)	58 (14)	<0.05^5^
**EF ≤ 35% (%)**	7	4	11	<0.05^5^
**NYHA II (%)**	11.9	13.0	6.5	
**NYHA III (%)**	59.9	54.3	56.3	<0.05^5^
**NYHA IV (%)**	28.2	32.6	37.2	
**Operation Characteristics**				
**Three–vessel Disease (%)**	61.1	59.7	58.6	NS
**Left Main Disease (%)**	13.1	16.6	14.1	NS
**Number of Grafts**	3.39	3.38	3.35	NS
**Use of Arterial Grafts (%)**	86.3	83.4	81.0	NS
**Concomitant Operation (%)**	6.1	3.3	7.6	NS
**Perfusion Time (SD)**	107.0 (36.6)	108.0 (58.8)	112.3 (34.2)	NS
**Urgent or Emergency Operation (%)**	26.7	29.9	33.5	NS

Abbreviations: *MI* myocardial infarct, *TIA* transient ischemic attack, *EF* ejection fraction, *NYHA* angina pectoris symptoms according to New York Heart Association, *CHD* coronary heart disease, *LM* left main, low HDL cholesterol: men < 1.03 mmol/l, women < 1.3 mmol/l, elevated triglycerides: ≥ 1.7 mmol/l, NS = not significant, NA = not applicable.

^1^Unadjusted p < 0.01 by the pooled t-test for the comparison DM-/MetS+ against DM-/MetS-.

^2^Unadjusted p < 0.01 by the pooled t-test for the comparison DM+ against DM-/MetS-.

^3^Unadjusted p < 0.001 by the pooled t-test for the comparison DM+ against DM-/MetS+.

^4^Unadjusted p < 0.001 by the pooled t-test for the comparison DM+ against DM-/MetS-.

^5^Unadjusted p < 0.05 by the pooled t-test for the comparison DM+ against other groups.

Non-diabetic patients with MetS had by definition more often metabolic abnormalities than those without MetS (Table 1). Similar findings were obtained in patients with diabetes, of whom 82% had MetS. Non-diabetic patients without MetS had more often normal renal function than others (P < 0.01). Diabetic patients were older having more angina pectoris and peripheral arterial disease than other patients (Table 1). Smoking habits and the prevalence of hypercholesterolemia did not differ between the three groups.

### Overall survival

**Figure 2:**
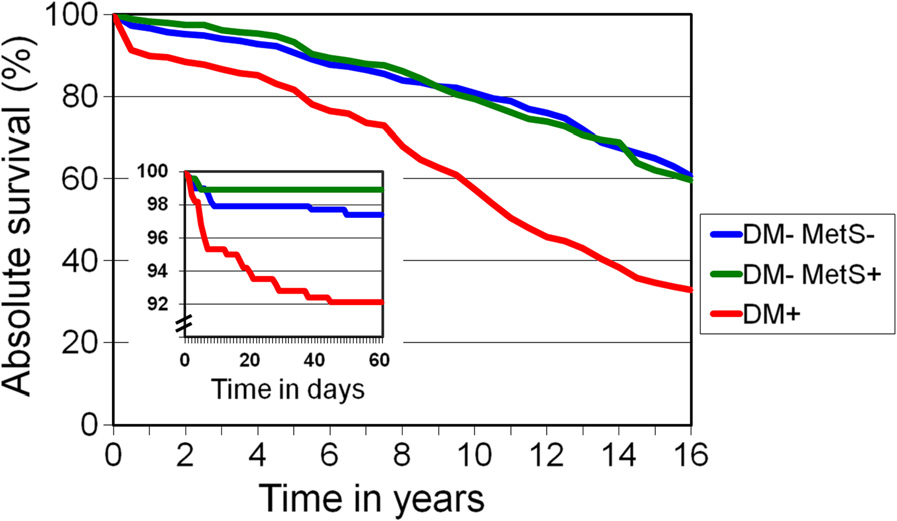
**Postoperative absolute survival of patients with neither diabetes nor metabolic syndrome (DM-/MetS-), patients with only metabolic syndrome (DM-/MetS+) and patients with diabetes regardless of metabolic syndrome (DM+) during the 16-year follow-up.** Comparison between DM-/MetS- and DM-/MetS+: NS Comparison of DM+ against both groups: P < 0.001. Follow-up of the first two months is shown in the small inset (n = 945).

No patients were lost during the 16-year follow-up. The overall postoperative survival is presented in Figure 2. DM was associated with significantly unfavourable prognosis compared to the non-diabetic patients in the two other groups (both p < 0.001). The difference in survival was already seen during the course of the first two postoperative months, where a total of 35 out of 945 patients (3.7%) died. Among non-diabetic patients there was no difference between subjects with or without MetS.

### Long-term survival against background population

**Figure 3:**
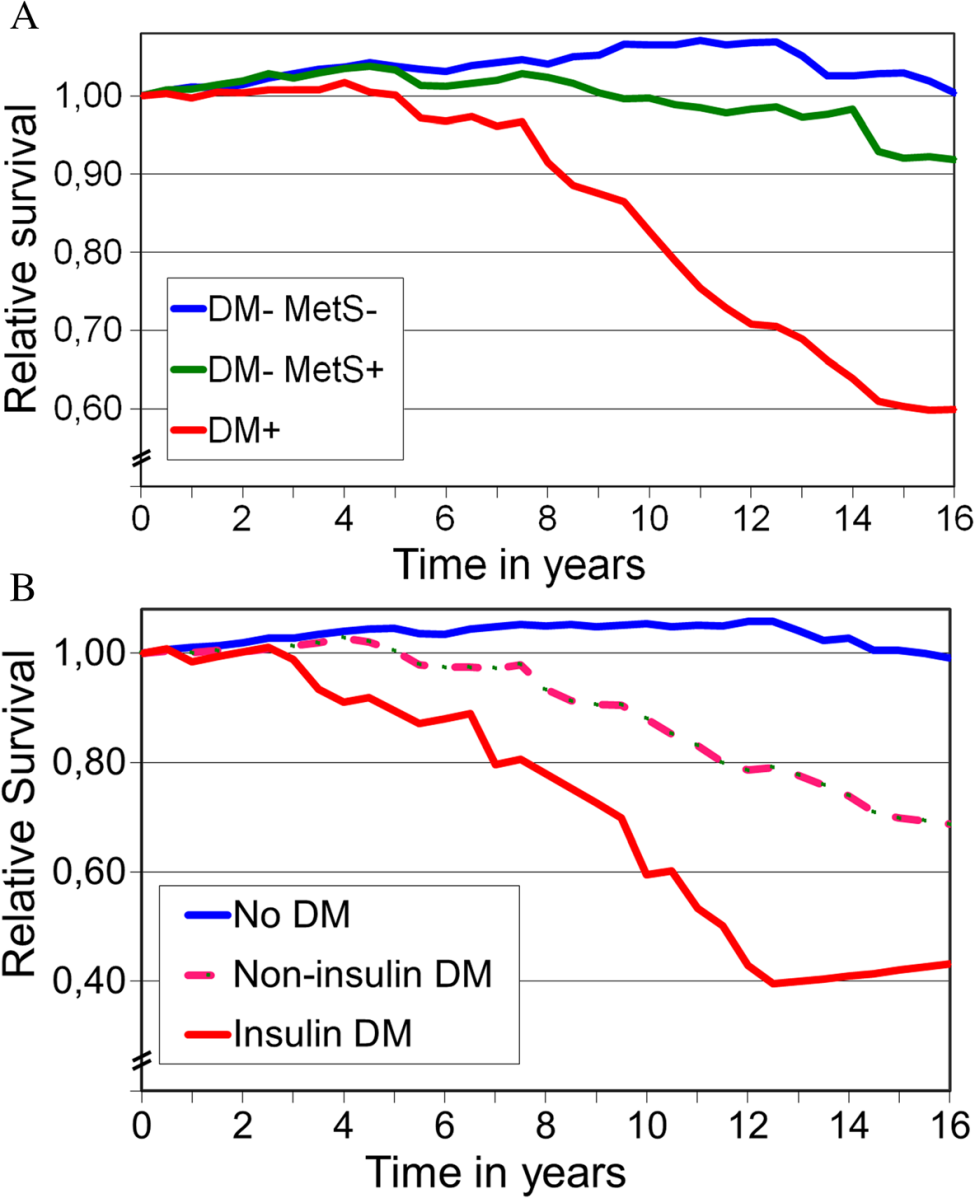
**The actual survival 2 months–16 years postoperatively compared to that of the age-and sex-matched Finnish population (relative survival 1.00, n = 910). A**. DM-/MetS- = patients with neither diabetes nor metabolic syndrome. DM-/MetS+ = patients with only metabolic syndrome. DM+ = diabetic patients. **B**. No DM = patients without diabetes. Non-insulin DM = diabetic patients without insulin treatment. Insulin DM = diabetic patients treated with insulin.

The analysis between 2 months and 16 years revealed that of the 910 patients alive two months postoperatively 432 (47.5%) had died. Figure 3A presents the relative survival of the three study groups matched by age, gender and calendar year against their respective Finnish background populations (relative survival = 1.00). After the perioperative phase, the 16-year relative survival of DM-/MetS- patients was 1.037, (95% CI, 1.026 to 1.048; < 0.0001) compared to that of the background population. For the first 10 postoperative years the relative survival was 1.033 (*95*% CI, 1.021 to 1.045; p < 0.0001).

### Metabolic syndrome and long-term survival

In non-diabetic patients with MetS the long-term survival between 2 months and 16 years was poorer than among patients without MetS (P < 0.0001). However, during the same period the relative survival of DM-/MetS+ patients resembled that of the matched background population (relative survival, 0.998; 95% CI, 0.978 to 1.018; NS).

During the first 10 postoperative years, the relative survival of DM-/MetS+ patients was 1.8% better than that of the matched background population (relative survival, 1.018; 95% CI, 1.010 to 1.026; p < 0.0001) but subsequently deteriorated thereafter (Figure 3A).

### Diabetes mellitus and long-term survival

In the postoperative period of 2 months to 16 years, the survival of patients with DM was significantly inferior compared to the background population (relative survival, 0.86; 95% CI, 0.82 to 0.90) and to both non-diabetic patient groups (all p < 0.0001; Figure 3A). DM patients on insulin therapy had poorer survival than those with not on insulin (p < 0.01, Figure 3B).

Among patients with DM, MetS seemed not to affect postoperative survival (2 months-16 years) after adjustment for age, gender and time (absolute survivals, 0.39; 95% CI, 0.28 to 0.50 for DM+/MetS+ and 0.36; 95% CI, 0.10 to 0.62 for DM+/MetS-, NS).

### Causes of death

Cardiovascular and non-cardiovascular death rates were 17.1% and 20.5% for DM-/MetS-, 18.3% and 21.5% for DM-/MetS+ and 38.7% and 28.1% for DM+ patients, respectively. Further, out of all deaths the corresponding proportions of cardiovascular deaths were 45.4%, 45.9% and 56.1%, respectively (p < 0.0001 between DM-and DM+ patients). Stroke was reported as a cause of death in only five patients.

### Best predictors of long-term prognosis

Bayesian multivariate analysis revealed together six variables to predict clinical outcome (2 months to 16 years) in relation to the national background population, *i.e.* age, diabetes, left ventricular ejection fraction, BMI, perfusion time during the CABG and peripheral arterial disease. Using this model, the measure of agreement (kappa = 0.45) was moderate.

## Discussion

To the best of our knowledge this study is the first in which the survival of non-diabetic and diabetic CABG patients was compared to that of matched background populations.

Our principal finding was that the long-term prognosis of patients with neither diabetes nor metabolic syndrome was better than that of the age-, sex-and time-matched background population. Further, in non-diabetic patients the presence of metabolic syndrome reduced long-term survival after CABG when compared to those without MetS. However, the mortality of MetS patients was not significantly inferior to that of the matched background population, which might reflect the careful postoperative follow-up and treatment of known risk factors of operated MetS patients.

Not surprisingly, DM was associated with significantly increased intermediate-and long-term mortality. Survival was even shorter if the diabetes treatment strategy included insulin compared to patients without insulin therapy. MetS *per se* did not impair survival among diabetic patients.

MetS, glucose intolerance and insulin resistance form together complex relationships, whose interacting pathophysiological roles are not yet fully understood. Insulin resistance leads to glucose intolerance, if pancreatic compensation processes are incomplete [21]. Glucose abnormalities with or without diabetes predict cardiovascular events and mortality [22,23]. Insulin resistance is also associated with MetS and its components [24]. Further, MetS has been shown to worsen the prognosis of coronary heart disease patients [25]. However, until recently no studies have specifically examined the prognostic significance of MetS for longer than 10 years after CABG.

Two studies have yielded different results regarding the impact of metabolic syndrome [8,9]. In the BARI trial no significant difference was seen in the death-rate or MI during the 10-year follow-up between patients with and without MetS [9]. In another study of CABG patients MetS predicted inferior outcome in the group of non-diabetic patients [8]. An increase in both all-cause and cardiac mortality became apparent approximately 10 years after surgery. The detrimental effect of MetS on survival was more marked in non-DM patients than in DM patients [8]. Our data are in concordance with and expand these findings.

Diabetes is associated with impaired outcome after CABG [26-28]. However, relation of insulin treatment on the long-term prognosis after CABG has been controversial [14,15,29]. Thourani and Alserius with their co-workers found independently that diabetic patients had a poorer 10-year prognosis than non-diabetic patients [14,29]. In both studies insulin-treated patients with diabetes had the poorest 10-year survival rate. Also, in a 10-year follow-up of CABG patients Mohammadi and colleagues found insulin-treated DM to be an independent risk factor for long-term cardiac mortality [15]. However, in DM patients not on insulin therapy, the cardiac-specific survival was similar to that observed in non-diabetic patients [15].

Our results are in accord with the findings of Thourani and Alserius, suggesting inferior survival of DM patients on insulin compared to those without insulin or to non-diabetics. Insulin treatment might suggest more severe and advanced diabetes in this cohort.

We found, that the excessive death rate of diabetics was mostly but not completely related to cardiovascular causes. Our results conform to the idea that long-lasting DM is associated with a generalized vascular disease which is characterized by impaired vascular endothelial function and hypercogulation [14,29].

The strength of the present study is the reliability and scope of the national registers used, allowing 100% coverage of the mortality data of our patients [30]. Our follow-up includes all subjects operated, like those migrated in the country and one patient moved in the neighbouring country. The length of the postoperative follow-up was exactly 16 years allowing conclusions more valid than those based on a shorter follow-up. According to Finnish health care policy every citizen has access to health care mainly financed by taxes. So in the background population such social factors, like a proportion of non-secured citizens, did not affect our results [31].

Certain limitations should also be considered. Firstly, the perioperative data were collected retrospectively [16]. Even though information was completed with patient questionnaires and direct contacts, 16 (1.7%) out of the original sample of 961 patients were excluded by reason of insufficient data. Secondly, we identified BMI-based obesity, not waist circumference, as a factor of the metabolic syndrome. Nevertheless, BMI is widely used in the modified definition of MetS and studies have demonstrated concordance between the definitions of MetS [7,18].

## Conclusions

In this long-term follow-up study patients with neither DM nor MetS had extremely good prognosis for at least 16 years when compared to the matched background population. Also the survival of patients with MetS but without DM had a good prognosis*.* Survival of DM patients started to deteriorate already short after the operation.

## Competing interests

The authors declare that they have no competing interests.

## Authors’ contributions

VH and AP designed the study. They acquired the final data and drafted the manuscript. MK carried out data maintenance and statistics. JL, MT and SV participated in the design and drafting of the manuscript. Other members of the W-CABG study group participated in their own hospitals in data acquisition. All authors read and approved the final manuscript.

## Supplementary Material

Additional file 1: Table S1 Univariate predictors of mortality. All variables presented here were taken into the Bayesian multivariate analysis. Click here for file
