# Supplementary material for: Associations of metabolic syndrome and diabetes mellitus with 16-year survival after CABG
Source: Cardiovasc Diabetol. 2014 Jan 22;13:25. doi: 10.1186/1475-2840-13-25 (PMC3914357; doi:10.1186/1475-2840-13-25)
Supplement: Additional file 1: Table S1 — Univariate predictors of mortality. All variables presented here were taken into the Bayesian multivariate analysis. [file 1475-2840-13-25-S1.doc]

Additional material

Table 2: Univariate predictors of mortality. All variables presented

here were taken into the Bayesian multivariate analysis.

**Preoperative Characteristics P-value**

Sex NS

Age p<0.0001

BMI group p<0.01

CV disease heritage p<0.01

DM p<0.01

Preoperative hemoglobin concentration p<0.001

Preoperative kidney funktion p<0.0001

S-Cholesterol p<0.001

S-HDL-cholesterol NS

S-Triglyserides NS

Obstructive pulmonary disease NS

Central vascular disease (mainly stroke) p<0.01

Periferal vascular disease p<0.0001

Hypertension p<0.01

Congestive heart disease p<0.0001

Atrial fibrillation p<0.05

Number of myocardial infarcts p<0.05

Earlier PCI NS

Earlier CABG NS

Ejection fraction p<0.001

Amount of preoperative cardiac medication p<0.0001

Preoperative lipid lowering medication p<0.05

Preoperative psychiatric medication NS

NYHA class p<0.001

**Characteristics of CABG**

Three-vessel disease p<0.01

Left main disease NS

Number of grafts p<0.01

Use of arterial grafts NS

Concomitant operation NS

Perfusion time p<0.0001

Urgent or emergency operation NS
